# Use of 3D printing to support COVID-19 medical supply shortages: a review

**DOI:** 10.2217/3dp-2020-0031

**Published:** 2021-07-13

**Authors:** Stephanie Ishack, Shari R Lipner

**Affiliations:** 1 New York University School of Medicine, NY 10021, USA; 2 Department of Dermatology, Weill Cornell Medicine, NY 10021, USA

**Keywords:** 3D printing, coronavirus disease 2019, COVID-19, epidemic, hydroxychloroquine, N95 respirator masks, personal protective equipment, PPE, respiratory tract infections, SARS-CoV-2, supply shortages, three-dimensional model, three-dimensional printing, ventilator

## Abstract

The novel coronavirus, COVID-19, created a pandemic with significant mortality and morbidity which poses challenges for patients and healthcare workers. The global spread of COVID-19 has resulted in shortages of personal protective equipment (PPE) leaving frontline health workers unprotected and overwhelming the healthcare system. 3D printing is well suited to address shortages of masks, face shields, testing kits and ventilators. In this article, we review 3D printing and suggest potential applications for creating PPE for healthcare workers treating COVID-19 patients. A comprehensive literature review was conducted using PubMed with keywords “Coronavirus disease 2019”, “COVID-19”, “severe acute respiratory syndrome coronavirus 2”, “SARS-CoV-2”, “supply shortages”, “N95 respirator masks”, “personal protective equipment”, “PPE”, “ventilators”, “three-dimensional model”, “three-dimensional printing” “3D printing” and “ventilator”. A summary of important studies relevant to the development of 3D-printed clinical applications for COVID-19 is presented. 3D technology has great potential to revolutionize healthcare through accessibility, affordably and personalization.

The COVID-19 outbreak was first reported in Wuhan, China in December 2019, resulting in a worldwide public health threat [1]. The race to obtain medical supplies reflects a global panic over a dwindling supply of N95 respirator masks, face shields, ventilators, testing kits and other personal protective equipment (PPE) [1–4]. Adequate production of PPE is essential during the COVID-19 pandemic to protect healthcare workers from viral transmission. 3D printing can be used to create intricate architectures to aid with these shortages. 3D printing is an integrated approach to robotic fabrication, using computer-aided design (CAD) systems to deposit layers of biomaterials (within external anatomy, within internal anatomy and replacement parts for devices) [5–10]. The success of a medical device is not only dependent on the type of biomaterial used for its fabrication but also on the structural integrity and quality (defect free) of the printing parts. Additive manufacturing technologies have opened new opportunities for manufacturing and production paradigms [9–13]. The primary advantage of using additive manufacturing is for on-demand and redistributed manufacturing to circumvent the supply chain disruption. Moreover, additive manufacturing allows for lower energy costs, reduced waste and is affordable. Ideal biomaterials should be readily printable, mechanically stable and biocompatible [5–7]. With ongoing materials research used in 3D technology, there is potential for innovative and cost-effective applications for addressing this current global crisis. Furthermore, the primary advantage of using additive manufacturing is for on-demand and redistributed manufacturing to circumvent the supply chain disruption. This review summarizes the key elements and advantages of 3D technologies that can be used to create 3D-printed tools to protect healthcare workers during the COVID-19 pandemic.

## 3D-printing techniques

### Extrusion-based printing

Extrusion-based printing utilizes print nozzles that extrude material by air pressure or mechanical force, with continuous printing in a layer-by-layer design for controlled and accurate deposition. Synthetic polymers that are commonly used in extrusion printing include, acrylonitrile butadiene styrene (ABS), polyurethane polyvinylpyrrolidone, polyvinyl alcohol and polylactic acid [9,10].

The most common type of extrusion-based printing utilized is fused-deposition modeling (FDM) [6–12]. FDM is fast, effective, and allows for easy integration with different CAD softwares. FDM uses thermoplastic filaments that pass through multiple heated printer nozzles and can therefore print multiple types of materials simultaneously [9,10]. Nylon, ABS, polylactic acid, polyvinyl alcohol, polycarbonate (PC) and polycaprolactone can be printed by FDM [5–10]. Furthermore, FDM can be utilized to build constructs in a timely manner with 3D accuracy and excellent mechanical properties [5–12]. Thus, FDM can be used to create customized patient and physician-specific medical devices, such as masks, face shields, ventilator valves that can be used during the COVID-19 pandemic.

### Material sintering

Material-sintering 3D technology is used to fuse powdered biomaterial into solid objects via physical (UV/laser/electron beam) or chemical (binding liquid) sources [14]. Common material sintering processes are called stereolithography (SLA) and selective laser sintering (SLS) [1–11].

SLA is the most often utilized to create prototypes layer by layer using photochemical processes to cross-link polymers, also called photopolymerization [14–16]. SLA is not only rapid and cost effective, but the versatility of 3D printing provides a myriad of clinical applications. Commonly used materials include resins, polyvinyl cinnamate, polyamide (PA), polyisoprene, polyimides and other photopolymers [14–16].

SLS uses a high-power laser beam to fuse the powdered materials in a layer-by-layer pattern to create an object. A high-power beam is controlled by CAD software and guides the printer to trace a cross section of the object onto the powder. The laser heats the powder either to just below its boiling point (sintering) or above its boiling point (melting), which merges the particles in the powder together into a solid form [17,18]. Thereafter, sequential layers of powder are fused together, and the process continues until the complete object has been printed.

**Figure 1.**
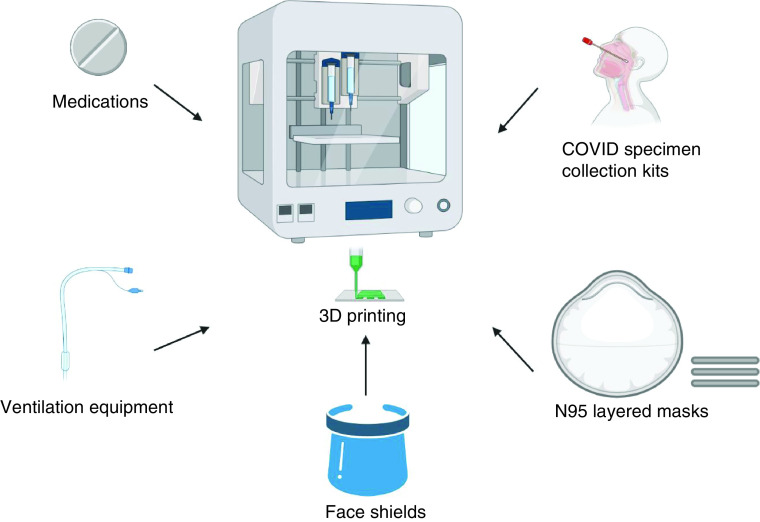
There are various medical devices that can be created via a 3D printer. Specific biomaterials can be used to create potential N95 3D-printed mask prototypes (layers to ensure effective filtration), face shields, ventilation equipment, COVID specimen collection kits and medications.

Both SLA and SLS have remarkable capability in the creation of customized medications and other tools to address the PPE shortage (Figure 1).

## Masks

N95 respirators masks are >95% efficient at filtering 0.3-μm airborne particles and require a fit test to ensure an adequate face seal [19–30]. The CDC currently recommends N95 masks for healthcare workers caring for COVID-19 patients [29]. 3D facial laser scanning combined with 3D printers can be used to scan exact facial parameters and create customized N95 face seals for improved mask comfort and fit. Important research studies relevant to the development of 3D-printed clinical applications for COVID-19 are shown in Table 1. Potential research studies relevant to the development of 3D-printed clinical applications for COVID-19 are shown in Table 2.

**Table 1.** Research studies relevant to the development of 3D-printed clinical applications for COVID-19.

Medical device	Author	Application	Materials	3D-technology fabrication process	Ref.
N95 mask	Swennen *et al.*	N95 mask-respirator mask	Two reusable 3D-printed components (a face mask and a filter membrane support) and two disposable components (a head fixation band and a filter membrane)	Additive manufacturing, SLS 3D printer	[30]
	McAvoy *et al.*	N95 filtering facepiece 51 respirators (FFRs or ‘masks’)	Halyard H600 sterilization wrap	Additive manufacturing, SLS 3D printer	[31]
	Moore-Imbrie *et al.*	3D-printed mask adaptor	Silicone, multipurpose polyurethane resins	A carbon M2 3D printer	[32]
3D-printed face shield	Wesemann *et al.*	3D-printed face protective shield	PET	Fused deposition modeling printer	[33]
	Sapoval *et al.*	3D-printed face protective shield	PVC sheet	Fused deposition modeling (material extrusion technique)	[34]
	Lemarteleur *et al.*	3D-printed face protective shield	Polylactic acid, polyethylene terephthalate glycol or acrylonitrile butadiene styrene	Fused deposition modeling (material extrusion technique)	[35]
COVID-19 specimen collection kit	Sananès *et al.*	NP swabs	Thermoplastic polymer, ABS	PolyJet technology, fused deposition modeling	[36]
	Ford *et al.*	NP swabs	Surgical guide version 1 resin	Stereolithography	[37]
	Oland *et al.*	Medical lattice swab	Resin (the “lattice swab”)	Carbon digital light synthesis printing	[38]
	Oland *et al.*	Direct-printed NP swab	Biocompatible photocurable resin (the “origin KXG”)	Additive mass manufacturing	[38]
	Arjunan *et al.*	Auxetic nasopharyngeal swabs	Photopolymer	3D design only	[39]
Ventilation Equipment	Ayyıldız *et al.*	Splitter for use of a single ventilator	Acrylic resin	PolyJet technology	[40]
	Dhanani *et al.*	AMBU bag	PLA plastic gears	Rapid prototyping technologies (3D printing and 2D cutting)	[41]

ABS: Acrylonitrile butadiene styrene; AMBU: Artificial manual breathing unit; FFR: Filtering facepiece respirator; NP: Nasopharyngeal; PET: Polyethylene terephthalate; PLA: Polylactic acid; PVC: Polymerizing vinyl chloride; SLS: Selective laser sintering.

**Table 2.** Potential research studies relevant to the development of 3D-printed clinical applications for COVID-19.

Medical device	Application	Materials	3D-technology fabrication process	Research design/key findings	Ref.
N95 mask	Mask face seal	Acrylonitrile butadiene styrene	FDM	Improved contact pressure with 3D-printed face seal compared with use of 3M© 8210 N95 FFR respirator masks	[27]
	Respirator mask	Silicon	RP	Printed silicon masks through digital modeling prototyped by RP	[21]
	Respirator mask	SEBS + PP	ME3DP	Printed biocompatible thermoplastic elastomeric materials from PP/SEBS compositions based on a facile blending strategy	[30]
	Respirator mask	PP	3D melt electrospinning printing	Sequential fiber layering achieved with a fiber diameter of 16.4 ± 0.2 μm; direct-writing of polypropylene	[42]
Face shield	Face shield mask	Polycarbonate, polyethylene, polyester, polyvinyl chloride, polyethylene terephthalate, polylactic acid	FDM	3D-printed transparent face shield	[31]
COVID-19 specimen collection kit	NP/OP swabs	Polyethylene terephthalate (dacron), polyester mesh, nylon flocked swabs	FDM	Can produce 3D-printed NP/OP prototypes that are strong and flexible	[43]
	NP/OP swabs	Surgical guide resin	3D-printed test resin via SLA laser printing	Can produce NP/OP flexible swabs	[44]
Ventilation equipment	Ventilator valves	Polyamide, polysulfone, polycarbonate, silicone rubber, nylon and polyamide 12 (PA12)	Filament extrusion system	Can create valve has very thin holes and tubes, smaller than 0.8 m	[45,46]
	Mechanical BVM adaptors	PVC and polyethylene valve	Filament extrusion system	Can create adapters and valves to connect the AMBU bag to the face mask	[47]
	Venturi mask	PVC	Filament extrusion system	Can design custom masks that allow high oxygen flow of a known oxygen concentration to patients	[47]
	LMA	PVC, silicone	Filament extrusion system, RP	Can design custom adapters/ valves to connect the LMA to an oxygen source or an expiration tube; can also produce a mold of a silicon mask via RP	[47]
	Tracheal tube	PVC, silicone	Filament extrusion system, RP	Can create custom adapters/valves to connect the LMA to a regulated oxygen flow source (from ventilator to patient) or an expiration tube (from patient to ventilator)	[48]
	NRB	PVC	Filament extrusion system	Can produce custom adapters and valves to connect the mask to the oxygen source	[48]
	Mask or helmet for CPAP ventilation	Face mask – PVC, polycarbonate; face seal – silicon; polyurethane	Filament extrusion system, DOD 3D printing	Can produce custom adapters/valves connected to CPAP mask/helmet to oxygen source; can produce the clips/attachments that holds the mask pressed onto face	[46]
	Ventilator valves	Polyamide	Polymer-laser powder/SLS	Can be used to melt and fuse a powder together to build up layers of an object	[45,46]
Medication	3D-printed tablet	PVA filament + fluorescein	FF 3DP	Successful fabricating personalized-dose medicines or unit dosage forms with controlled-release profiles	[49]
	Modified-release drug-loaded tablets	PVA filaments + (IBD), 5-aminosalicylic acid (5-ASA, mesalazine) + 4-aminosalicylic acid (4-ASA)	FF 3DP	Successful results show that it is possible to tailor oral drug dosage and modified-release formulation	[50]
	Drug-loaded tablets	PEGDA + diphenyl(2,4,6-trimethylbenzoyl) phosphine oxide + 4-aminosalicylic acid (4-ASA) + paracetamol (acetaminophen)	Stereolithography	SLA 3DP technology allows the manufacture of drug-loaded tablets with specific extended-release profiles	[51]
	Pharmaceutical bilayer tablets	Hydroxypropyl methylcellulose (HPMC 2208) + (Methocel™ K100M Premium) + PAA (Carbopol^®^ 974P NF) + MCC (Pharmacel^®^ 102) + SSG (Primojel^®^)	Gel extrusion	Successful drug release through a hydrated HPMC gel layer	[52]
	3D-printed medicine	Kollicoat IR (75% PVA + 25% polyethylene glycol copolymer) + Eudragit L100-55 (50% methacrylic acid and 50% ethyl acrylate copolymer) + paracetamol (acetaminophen)	SLS	Demonstrated the suitability of SLS printing technique using medical powders and lasers for manufacturing	[17]
	Personalized 3D-printed drugs	Hydrogel + PEG + hydroxypropyl methylcellulose + poly acrylic acid + aspirin	Coaxial needle extrusion	Print-active pharmaceutical ingredients; create combinations of controlled dosing of drugs; personalized medication	[53]

ABS: Acrylonitrile butadiene styrene; AMBU: Artificial manual breathing unit; BVM: Bag valve mask; CPAP: Continuous positive airway pressure; DOD: Drop-on-demand; FDM: Fused-deposition modeling; FF 3DP: Fused-filament 3D printing; LMA: Laryngeal mask airway; MCC: Microcrystalline cellulose; ME3DP: Material extrusion 3D printing; N95 FFR: N95 filtering facepiece respirator; NRB: Nonrebreather mask; OP: Oropharyngeal; PAA: Poly(acrylic acid); PEG: Polyethylene glycol; PEGDA: Polyethylene glycol diacrylate; PP: Polypropylene; PVA: Polyvinyl alcohol; PVC: Polyvinyl chloride; RP: Rapid prototyping; SEBS: Styrene-(ethylene-butylene)-styrene; SLA: Stereolithography; SLS: Selective laser sintering; SSG: Sodium starch glycolate.

### 3D imaging

N95 respirator masks are typically manufactured in small and medium sizes only and may not match perfectly with individual head and facial parameters. An imperfect fit may be uncomfortable for the user, and more concerning is that the mask is ineffective in blocking contaminated air. Therefore, comfort and fit are two important parameters for respirator design and development. Cai *et al.* created face seal prototypes with acrylonitrile butadiene styrene plastic using a FDM 3D printer. Anthropometric data of the chin arc, jawline, face and nose shape and lengths, and nose protrusion measurements were collected via 3D laser scanning method to create a tailored seal [27]. Three test subjects showed improved contact pressure compared with a 3M© 8210 N95 FFR respirator masks [27]. Moreover, a personalized mask can be designed to account for the presence of facial hair length and density.

Table 3 displays 3D-imaging technologies and key findings of several studies used to create N95 mask seals [19–28]. Han *et al.* successfully developed respirator prototypes via digital face modeling and used rapid prototyping to print silicon respirators [21]. Niezgoda *et al.* used a stereophotogrammetry technique to collect 3D facial geometry of subjects with and without wearing a molded, cup-shaped N95 filtering facepiece respirators [23]. Standard size flat fold and cup-shaped N95 filtering facepiece respirators had significantly different seal pressures (p < 0.01). Thus, imaging technologies provide recommended construction of optimally fitted respirator seals and masks that can be worn by healthcare workers treating COVID-19 patients.

**Table 3.** Displays the 3D-imaging technology and key findings of several studies used to create N95 mask seals.

3D Technology	Research design/key findings	Ref.
3D laser-scanning method	Used a high-precision hand scanner, zgHandScan H100 captured up to 480,000 points per second	[27]
3D face scanner	Created three differently face models (small, medium and large) using a 3D face scanner and developed three corresponding respirators through digital modeling; designed three silicon respirator models via rapid prototyping method	[21]
SPG	Used to collect 3D images of subjects with and without wearing a molded, cup-shaped N95 FFRs; recorded geometric data from photographic images	[23]
3D face scanner	Used a minimal set of landmarks to derive contact area for a half-face mask on individual human faces	[22]
3D real-time surface-pressure mapping system	Mapping system simulated contact between six N95 FFRs and five digital headform models to understand contact pressure	[19]
MATLAB computer-based algorithm	Developed a computer-based algorithm to determine the contact area between the headform models and N95 FFRs	[20]
3D laser-scanning method	Recorded contact pressure between a respirator and a digital headform	[25]

N95 FFR: N95 filtering facepiece respirator; SPG: Stereophotogrammetry.

### 3D printing

Standard N95 masks consist of filtration material consisting of electrostatic nonwoven polypropylene (PP) fibers which are semi-rigid, light and fatigue resistant. The semi-crystalline structure may cause significant damage and distortions of the 3D-printed parts upon cooling thereby making 3D printing difficult; however, a combination polymer can help to support the creation of N95 masks. Material extrusion 3D printing was used to design a 3D-printable thermoplastic elastomeric material from a blend of PP and styrene-(ethylene-butylene)-styrene (SEBS) [30]. PP is commonly used for various industrial applications due to its processability, printability, recyclability and durability. SEBS is a thermoplastic with low processing temperature, good elasticity and low distortion during Material extrusion 3D printing [29,54]. Therefore, the PP/SEBS blend provides better printability and flexibility for N95 mask design. Furthermore, the thermoplastic elastomer ratio allows for customizing the durability and elasticity of the biomaterial for better-fitted 3D masks [27–30,54,55].

Setz *et al.* investigated the morphology of the PP/SEBS blend via scanning electron microscopy and transmission electron microscopy [56,57]. Using scanning electron microscopy, cryofracture surfaces did not dislodge any particles, thereby confirming good compatibility between SEBS and PP. Transmission electron microscopy micrographs demonstrated that SEBS diffuses into PP resulting in biomaterial crosslinking and contributing to increased interfacial strength and elongation.

Haigh *et al.* demonstrated use of PP microfibers in a 3D melt electrospinning printer. Several sequential fiber layers of material were printed to accurately obtain the 3D form with fiber diameters as small as of 16.4 ± 0.2 μm [42]. Thus, 3D-printing procedures may allow for the creation of biocompatible N95 masks that are comparable to industrial manufacturing brands. Table 1 displays 3D-printing techniques, materials and applications for N95 3D-printed masks.

McAvoy *et al.* developed a frame for N95 masks, using biomaterials and 3D-printing technologies [31]. The authors designed a mask frame consisting of two 3D-printed side pieces, malleable wire links that users press against their face, and cut lengths of elastic material that wrap around the head to hold the frame and mask in place. The masks passed qualitative fit testing varying from 48 to 92% (depending on mask model and tester). For individuals for whom a mask passed testing, 75–100% (average: 86%) also passed testing with a frame holding the mask in place [31].

Moore-Imbrie *et al.* developed a solution for the COVID-19 N95 mask shortage by designing a mask adaptor that maintains the N95 seal standard. Several designs were 3D-printed and optimized based on filter surface area, seal efficacy, and N95 respirator multiplicity; the final design was a 3D-printed soft silicone base and a 3D-printed rigid cartridge to seal one-quarter of a 3M 1860 N95 mask [32]. All participants passed computerized qualitative mask fit testing (6/6). The PortaCount Respirator Fit Tester was used to measure the concentration of microscopic particles outside the mask and leakage inside the mask. The ratio of these two numbers is the fit factor that is used for assessment of standard N95 mask seal. The overall fit factor measured was 148 ± 29, with 100 as the standard pass level for an 1860 N95 mask. In addition, the open-source files are publicly available for other researchers to utilize.

Swennen *et al.* produced 3D-printed personalized masks by utilizing multiple filtration units [30]. These individualized 3D masks consist of two 3D-printed PA composite components (a face mask and a filter membrane) and disposable components (a head fixation band and a filter membrane). The authors used CAD to measure the face mask based on individual facial scans [30].

## Face shields

Face shields are PPE devices used to protect facial areas and associated mucous membranes (eyes, nose, mouth) from sprays and splatter of body fluids. Face shields offer an additional layer of protection from COVID-19 droplets, with sufficient space room to wear a protective N95 mask underneath. Face shields are comprised of three components, a robust headband, a durable plastic shield and an elastic band. PC, polyethylene, polyester, polyvinyl chloride, polyethylene terephthalate, polylactic acid and other synthetic polymers are commonly used to make surgical face shields and can be 3D printed via FDM [58,59]. Table 1 shows the different biomaterials and 3D technology that can be used to construct a face shield design.

There are several advantages of 3D printing for manufacturing face shields. Due to their simple design, 3D printers can easily provide thousands of face shields per day. Additionally, the biomaterials used to create these shields can be easily sanitized for repeated wear if necessary. Specifically, antimicrobial polymers allow engineers to prototype face shields and other critical medical devices [59].

## Ventilation equipment

Globally, one of the biggest challenges amid the COVID-19 crisis is when the number of critical care patients exceeds the available medical infrastructure. Based on data from Wuhan, China, 56% of COVID-19 patients that were admitted to the intensive care unit (ICU) required noninvasive ventilation (NIV) and 76% required further orotracheal intubation and invasive mechanical ventilation [45,46]. Therefore, ventilation devices are in high demand during the COVID-19 pandemic.

## Ventilator valves/adaptors

Ventilator valves are attachments used to deliver oxygen at fixed concentrations for patients with acute respiratory distress, including COVID-19 patients. 3D-printing technology can be used via a filament extrusion system or a polymer-laser powder/SLS bed fusion process to print single-use valve sets [46,60]. 3D printers can be used to design the different elements of the valve using biomaterials such as PA, polysulfone, PC, silicone rubber, nylon and PA 12 (PA12) [45,46,59–61]. Furthermore, these disposable valves eliminate time-consuming sterilization.

## Mechanical bag valve mask

3D-printed emergency-respiration custom adapters and valves can be used to connect to mechanical bag valve mask (BVM) or artificial manual-breathing unit bags (AMBU) [46,62–64]. This mechanical BVM is meant for short-term emergency ventilation of COVID-19 patients while more critical patients would require long-term (>2 weeks) ventilation with controlled settings based on humidification, oxygen, filtration and pressure adjustments. One major advantage is that 3D-printed respirator adapters and valves are scalable with a production rate between 50 to 100 units per day [62–64]. It is also possible to add layers of automation sensors and customized regulation of air pressure and flow allowing for disease-specific and patient-tailored respiratory support implementations [63]. Moreover, these masks are very well-fitted to the user’s face which is particularly important to prevent emission of aerosols. Dhanani *et al.* performed *in vivo* and *in vitro* testing in pigs using a 3D modular ventilator [41]. The AMBU bag was connected to a wall oxygen source using a flow meter. The authors demonstrated comparable mechanical efficiency of the test ventilator compared with a standard ventilator [41]. The 3D ventilator is low cost and can be rapidly produced, but limitations include lack of data on plateau pressure (alveolar pressure) and positive end-expiratory pressure.

## Noninvasive ventilation

NIV therapy or continuous positive airway pressure uses face masks, nasal masks or mouthpieces to provide both oxygenation and ventilation support. NIV has been proposed for treatment of less-severe COVID-19 patients that do not require ventilators given the increasing demand for ICU beds for critically ill patients during this pandemic [41,61–64]. The WHO and the CDC recommend that NIV should be utilized in a negative-pressure isolation room for patients [63].

Makowski *et al.* developed customized respirators according to the anthropometric dimensions via 3D-scanning and -printing techniques [44]. These measurements were detected using a hand-held 3D scanner and the digital model of the facepiece was matched to the user’s face via CAD software. Thereafter, SLS was used to print tailored facepieces from thermoplastic polyurethane [63]. These respirators were very well-fitted and did not cause any facial imprints or contact dermatitis. The application of 3D facial scanning and printing techniques for designing and fabricating customized facepieces are a viable choice for development of respiratory protective devices, such as an NIV mask for COVID-19 patients. Some advantages of NIV include, less intensive monitoring and more efficient use of scarce medical resources like ICU beds [41,62–64]. Table 1 displays 3D-printing techniques, materials and applications for ventilation equipment.

## COVID-19 specimen collection kit

Nasopharyngeal (NP) swabs are flexible rods with bristled ends that are inserted into the nasal cavity to sample cells and mucus. Oropharyngeal (OP) swabs are used to collect specimens by swabbing the patient’s posterior pharynx and tonsillar area. Creating 3D-printed NP and OP test swabs would help increase COVID-19 testing capacity for patients worldwide. Testing swabs can be made from a flexible polymer, like dacron, nylon flocked, rayon, polyester or surgical guide resin, with customized formulations resulting in a wide range of mechanical, optical and thermal properties [65–67]. Moreover, swab bud lattice fibers can be made in customized fashion using 3D engineering [44,65].

An ideal NP and OP swab should have: efficient capillary hydraulics between the brush strands allowing to maximal absorption, a tip with perpendicular brush-like texture allowing the flocked nylon to efficiently dislodge and collect cells and mucus and a tip with an open lattice structure, allowing rapid automatic elution that releases the sample immediately when immersed in viral transport medium [65]. In addition, different sized (small, medium, large) nasal swabs can be printed for variations in nostril size to minimize patient discomfort. Table 1 shows the different biomaterials and 3D technology that can be used to construct NP and OP swabs.

Oland *et al.* performed a clinical validation study on 3D-printed NP swabs for the diagnosis of COVID-19 [38]. Seventy adult patients (37 COVID-19 positive and 33 COVID-19 negative) underwent consecutive diagnostic reverse transcription PCR testing with a flocked swab followed by one or two 3D-printed swabs. The ‘lattice swab’ demonstrated 93.3% sensitivity and 96.8% specificity and the ‘origin KXG’ demonstrated 83.9% sensitivity and 100% specificity. Thus, the authors concluded that 3D-printed NP swab results have high concordance with the control swabs [38].

Arjunan *et al.* developed 3D-printed auxetic NP swabs with the aim of reducing patient pain and discomfort [39]. These specific swabs can shrink under axial resistance thus allowing the swab to navigate through the nasal cavity with significantly less stress on the surrounding tissues [39].

One of the major advantages of using 3D technology to print NP and OP swabs is a production rate of 2000–3000 a day [66]. Another advantage is that synthetic swabs have a more effective sample release process when placed into a culture medium.

## Medications

3D technology such as fused filament, powder extrusion, gel extrusion, SLS and SLA allows for fabrication of printed medications for COVID-19 patients that are in short supply. On 31 March 2020, the US FDA added the antimalarial drugs, hydroxychloroquine and chloroquine, potential treatments for COVID-19, to its shortage list due to increased demand [38,65–67]. In addition, there has been an exponential increase for the antiviral drug, remdesivir, which is also in limited supply [49–52,68,69].

Hsiao *et al.* reported applications and challenges in applying 3D-printing technology to oral solid dosage forms production [70,71]. They noted that since 2018, other studies have shown multicomponent controlled-release polypills and custom capsule devices for sustained drug release, such as SLS, direct-powder extrusion and electrohydrodynamic printing [47,48,72–74]. They noted that many of repurposing drug candidates for COVID-19 have poor aqueous solubility and that oral administration of these drugs would need specific bioavailability enabling formulations, such as amorphous solid dispersion, for drug efficacy. Since specific formulations are necessary for anti-viral drugs, FDM printing and amorphous solid dispersion using hydrophilic polymers could be suitable [70]. Some limitations in these approaches are the high drug melting point that is needed for printing [70].

Multicompartment and multilayer 3D printing can be used for fixed doses or combination of two or more anti-viral therapeutics for COVID-19. It would be important to embed drugs in 3D-printed dosage forms that would then provide a barrier for physical and chemical degradation. The major challenges in 3D-printed medication are the synergy between drug formation and for selection of 3D-printing technology (i.e., ink-jet powder). Moreover, scalability, expense and time are other challenges.

Fused-filament 3D printing is a versatile delivery system used to fabricate tablets containing drug doses customized to individual patients or specific drug-release profiles. Goyanes *et al.* created tablets that had excellent mechanical properties and little thermal degradation [53,75–92]. Furthermore, dissolution tests showed that release profiles were dependent on the drug-fill percentages. Therefore, FF 3DP can be an exceptional solution for fabricating personalized-dose medicines or dosages with controlled-release profiles for COVID-19 patients.

SLA has been used to create 3D-printed medicine in several reports [15,16,53,78–90]. In one study, SLA was used to fabricate drug-loaded tablets with modified-release characteristics [78]. The medications were successfully printed, and dissolution simulations of the GI tract showed that the 3D-printed drug release from the tablets was dependent on the drug formation, but independent of dissolution pH. Thus, SLA is a remarkable tool for manufacturing of drug-loaded tablets with distinct release profiles [78].

SLS have remarkable capability in the creation of customized medications and other tools to address the PPE shortage [17,18,81,82]. Specifically, Fina *et al.* used SLS to successfully produce printed pills that showed no evidence of drug degradation [17]. Although there are no specific antivirals or vaccines for treatment of COVID-19, several well-characterized drugs are being considered as therapies [2,3,83–86]. It is feasible to use 3D medication-printing technology to effectively and quickly print, remdesivir, chloroquine and hydroxychloroquine pills [2,49,50,52]. Thus, 3D medication printing has great potential within the pharmaceutical industry in general, but also in the optimization of supply and distribution chains to aid in the treatment of COVID-19 patients.

## Limitations

There are several potential challenges for the development and approval of 3D printing of medical devices during the pandemic. First, medical devices need to be highly regulated for safety and efficacy; in-house expertise is of particular concern. Second, standard safety and quality measures of 3D-printing labs must be optimized. In regards to the pandemic, medical centers that have partnerships between 3D-printing resources and hospitals would need to follow specific safety protocols. This includes sterilization processes using newly-printed medical devices. Third, intellectual property remains a concern and thus regulators and policy makers must establish partnerships. Some other top 3D-printing concerns include part quality (integrity, strength and aesthetics), costs of biomaterials, printers and other equipment and cost of pre- and postprocessing. Scalability is also a challenge because mass production might be limited due to printing times, which can be typically a few hours.

For 3D imaging, there are inherent limitations. Specifically, understanding the 3D-imaging technology for personalized facial scanning for PPE may be challenging however 3D-imaging tutorials may be available for healthcare facilities and there may be options for virtual facial scanning to print N95 masks.

The biggest potential bottleneck for 3D printing is lack of standardization and potential for low-quality products. Printing technologies lack universal standards and thus many manufacturers and scientists may encounter issues with quality, strength and reliability of products. Thus, an industry-wide standardization for 3D printing and manufacturing is necessary. In addition, printing hardware failure and irregular maintenance frequency are a bottlenecks that merit consideration.

## Conclusion

Amid the rapidly progressing COVID-19 outbreak, there have been PPE shortages globally. 3D-printing technology is well suited to address COVID-19-related shortages by creating several low-cost medical equipments from cost-effective and readily-available polymers. However, processing time, clinical testing and skills shortage are potential barriers to creating 3D-printed medical equipment. Synthetic polymers needed for 3D-printed PPE are exact or very similar in biomaterial composition to the standard manufacturing grade products (i.e., N95 respirator masks, mask filters, NP and OP swabs, ventilator adaptors). Thus, 3D technology has great potential to revolutionize healthcare through accessibility, affordability and personalization. While optimizing mitigation strategies, 3D-printing technology can be used to yield a variety of tools that front lines healthcare workers can use in the fight against the COVID-19.

## Future perspective

Although 3D printing offers significant contributions to the healthcare, there are still unanswered questions on regulations for point-of-care manufacturing. Premarket approval submissions and FDA approvals must meet certain requirements to be fully functional for use. Moreover, these applications may take time to complete testing for approval. Some of the important aspects that need to be considered include, intellectual property for medical parts, a validated manufacturing process that adheres to specifications and regulations and personnel and equipment that are readily available at facilities. As 3D printing is adopted more widely for various applications, regulatory oversight is necessary to ensure safety. During the pandemic, several innovative partnerships with universities rapidly produced medical devices, thus highlighting the possibilities for the future production. Importantly, several organizations have openly sourced their 3D printing, allowing people from all over the world to have access. This open-source model is an attempt for equitable standardization and democratic availability from a software perspective. Over the next 10 years, applications of 3D printing in medicine will continue to grow with the goal of improving patient diagnosis and treatment options, as well as, medical equipment for healthcare systems. This transformative technology has the capability to significantly impact medicine in the coming years.

**Executive summary**

- The global spread of COVID-19 has resulted in shortages of personal protective equipment leaving frontline health workers unprotected and overwhelming the healthcare system.
- 3D printing allows researchers and engineers to design potential applications for personal protective equipment for healthcare workers treating COVID-19 patients.
- There are major developments of 3D-printed clinical applications for COVID-19 that is illustrated in this article.
- 3D-imaging technology can be used to create N95 mask seals.
